# SHCBP1 Is Upregulated in Colon Adenocarcinoma and Promotes Tumor Cell Proliferation and Growth

**DOI:** 10.3390/curroncol33050295

**Published:** 2026-05-19

**Authors:** Yiren He, Qian Zhang, Xinyang He, Wenyong Wu

**Affiliations:** 1 The Fifth Clinical Medical College, Anhui Medical University, No. 81, Meishan Road, Shushan District, Hefei 230032, China; heyiren2007@yeah.net; 2 Department of General Surgery, Anhui No. 2 Provincial People’s Hospital, Clinical College of Anhui Medical University, No. 1868, Dangshan Road, Yaohai District, Hefei 230011, China; 3 Department of General Surgery, The Affiliated Provincial Hospital of Anhui Medical University, No. 17, Lujiang Road, Luyang District, Hefei 230001, China

**Keywords:** COAD, SHCBP1, proliferation, biological function, virtual screening

## Abstract

Colon adenocarcinoma (COAD) is a common malignancy with substantial morbidity and mortality, and the identification of new therapeutic targets remains essential for improving patient outcomes. In this study, we investigated SHC SH2-domain binding protein 1 (SHCBP1) in COAD through two complementary components with distinct evidentiary scopes. The first component comprised expression profiling, prognostic and methylation analyzes, bioinformatic characterization, and functional validation in vitro and in vivo. The second component comprised exploratory computational analyses, including predicted interaction network analysis and structure-based virtual screening. Public databases were used to analyze SHCBP1 expression, prognosis, and promoter methylation status. Co-expression and functional enrichment analyses were performed to explore the biological context of SHCBP1. In vitro and in vivo experiments were then conducted to evaluate the effects of SHCBP1 knockdown on tumor growth. SHCBP1 was significantly upregulated in COAD and was associated with poor patient prognosis. Promoter hypomethylation may contribute to its increased expression. Bioinformatic analyses suggested that SHCBP1 is associated with DNA replication and cell-cycle-related pathways. Experimental studies demonstrated that SHCBP1 knockdown suppressed cell proliferation and tumor growth. In the exploratory computational component, predicted interaction network analysis and virtual screening prioritized several in silico candidate interactions and two compounds with favorable predicted binding scores. These computational findings require independent biochemical and cellular validation. Overall, our findings suggest that SHCBP1 may represent a candidate biomarker associated with COAD proliferation and unfavorable prognosis, as well as a putative molecular target that warrants further validation.

## Simple Summary

Colon adenocarcinoma (COAD) is a common and life-threatening malignancy, and new molecular targets are needed to improve treatment. In this study, we investigated SHCBP1 in COAD using public-database analyses, tissue-level validation, and functional experiments. SHCBP1 was highly expressed in tumor tissues and was associated with unfavorable prognosis. Functional experiments showed that SHCBP1 knockdown inhibited colon cancer cell proliferation and suppressed tumor growth in vivo. We also included exploratory computational analyses, including predicted interaction-network analysis and structure-based virtual screening, to generate hypotheses for future mechanistic and pharmacological studies. Overall, our findings support a role for SHCBP1 in COAD cell proliferation and tumor growth and provide a basis for further investigation.

## 1. Introduction

Colon adenocarcinoma (COAD) poses a significant global health challenge due to its high incidence and mortality rates. Advances in treatment modalities have not substantially improved the 5-year survival rate, particularly in advanced or metastatic cases [1,2,3]. The pathogenesis of COAD involves complex interactions between genetic mutations, epigenetic alterations, and environmental factors, disrupting key oncogenic pathways and molecules [4]. Thus, identifying new molecular targets in COAD is important for developing targeted therapies and improving patient outcomes.

SHC SH2-domain binding protein 1 (SHCBP1) is a highly conserved scaffold protein that plays a crucial role in transmembrane receptor signaling [5]. It is widely expressed in human tissues, modulating signaling pathways and participating in various physiological and pathological processes [6]. SHCBP1 has been implicated in tumor progression in several cancers, including gastric [7,8], liver [9], pancreatic [10], esophageal [11], bladder [12], and breast cancer [13,14]. Notably, previous studies have generated both global and conditional Shcbp1 knockout mice and found that loss of Shcbp1 did not cause obvious defects in T-cell development; moreover, Shcbp1-deficient mice were viable and fertile. However, Shcbp1 deficiency reduced disease severity and improved survival in an experimental autoimmune encephalomyelitis model, suggesting that SHCBP1 may be dispensable for normal development but functionally relevant under pathological conditions [15]. Nevertheless, the expression patterns, functional mechanisms, and biological functions of SHCBP1 in COAD remain inadequately characterized.

In the present study, we investigated SHCBP1 in COAD through two complementary components. The first component focused on expression profiling, prognostic and methylation assessment, bioinformatic characterization, and functional validation in vitro and in vivo. The second component consisted of exploratory computational analyses, including predicted interaction network analysis and structure-based virtual screening, with the aim of generating hypotheses for future mechanistic and pharmacological studies. Our findings support an association between SHCBP1 and COAD cell proliferation and tumor growth, while the virtual screening analysis provides preliminary candidates for future pharmacological evaluation.

## 2. Materials and Methods

### 2.1. Study Framework

The work reported here is presented in two complementary components with different evidentiary scopes. The first component comprised expression, prognostic, methylation, co-expression, and functional analyses, together with experimental validation in vitro and in vivo. The second component comprised exploratory computational analyses, including predicted protein interaction modeling and structure-based virtual screening. The computational component was intended for hypothesis generation only and was not experimentally validated in the present study.

### 2.2. Analysis of Public Databases

To explore the *SHCBP1* expression and prognostic value in COAD, we conducted analyses utilizing publicly available databases, including ONCOMINE (https://www.oncomine.org) [16], TIMER (https://cistrome.shinyapps.io/timer/, accessed on 13 May 2026) [17], GEPIA2 (http://gepia2.cancer-pku.cn) [18] and the Human Protein Atlas [19] (The FPKM cutoff was set as 3.03) databases (https://www.proteinatlas.org). The UALCAN database (http://ualcan.path.uab.edu) was used to assess the *SHCBP1* promoter methylation patterns in cancerous and normal tissues. Methylation sites and prognostic implications of *SHCBP1* were identified using MethSurv database (CpG sites were selected from the HM450K array in COAD samples) (https://biit.cs.ut.ee/methsurv/, accessed on 13 May 2026) [20,21]. The *SHCBP1* co-expression profiles were obtained from the LinkedOmics database (http://www.linkedomics.org) and visualized as volcano plots and heatmaps [22]. These co-expressed genes were then used for Gene Ontology (GO) and Kyoto Encyclopedia of Genes and Genomes (KEGG) enrichment analyses. Additionally, GeneMANIA (https://genemania.org) [23] and STRING (https://cn.string-db.org) databases [24] were used to construct the protein–protein interaction (PPI) network and identify potential interaction partners of SHCBP1. Detailed URLs and versions are listed in Supplementary Table S1. Because these databases differ in cohort composition, normalization pipelines, and sample sizes, the results were interpreted as convergent supportive evidence rather than strictly interchangeable measurements.

### 2.3. Cell Culture and Lentiviral Infections

The NCM460, HT29, HCT15, HCT116, and CW2 cell lines were obtained from the American Type Culture Collection (Manassas, VA, USA) or Procell (Wuhan, China). These cell lines were, respectively, cultured in McCoy’s 5A, RPMI-1640, or DMEM medium, supplemented with 10% heat-inactivated fetal bovine serum and 50 U/mL penicillin/streptomycin solution, in a humidified incubator at 37 °C with 5% CO_2_. For lentiviral infections, specific shRNA sequences (Table S2) were individually cloned into the pLKO.1-puro vector (Addgene plasmid No. 8453) following a standard protocol. 293T cells in 10 cm dishes were transfected with psPAX2 (3 μg), pMD2.G (9 μg), and the gene knockdown plasmid (12 μg). Lentiviral supernatants collected 48 h post-transfection were filtered through a 0.45 µm strainer (Corning, NY, USA) and used to transduce target cells. At 72 h post-infection, cultures were switched to complete medium containing 2 μg/mL puromycin, refreshed every 48 h for 7 days. Cells were harvested directly for RT-qPCR and Western blot validation. Among the tested COAD cell lines, HCT116 cells were selected for most functional experiments because they showed relatively high SHCBP1 expression and stable transduction efficiency in our preliminary experiments.

### 2.4. Patients and Samples

Human colon adenocarcinoma tissue microarray sections (HColA160CS01), which comprised 80 paired tumor and adjacent normal tissue samples, were obtained from Shanghai Outdo Biotech Co., Ltd. (Shanghai, China). Ethical approval for the study protocols was granted by the company’s Ethics Committee under the approval ID: SHYJS-CP-170108. Paired COAD tumor tissues and adjacent non-tumor tissues were collected from patients who underwent surgical resection at our institution. All tissue samples were obtained with informed consent and were handled in accordance with institutional ethical regulations. Fresh tissue specimens were immediately frozen in liquid nitrogen and stored at −80 °C until use.

### 2.5. Real-Time qPCR

Total RNA was extracted using the TRIzol reagent, and subsequently reverse-transcribed to cDNA employing the PrimeScriptTM 1st Strand cDNA Synthesis Kit (Thermo Fisher Scientific, Waltham, MA, USA). Real-time qPCR was performed using a SYBR Green PCR kit (Applied Biosystems, Carlsbad, CA, USA) according to the manufacturer’s instructions. Relative gene expression was calculated using the 2^−ΔΔCt^ method. Primer details for SHCBP1 and GAPDH are available in Table S2.

### 2.6. Western Blot

Cell lysates were prepared with RIPA buffer, and protein concentrations were determined using the BCA Protein Assay Kit (Beyotime, Shanghai, China). Subsequently, 20 µg of each sample underwent SDS-PAGE on a 10% gel for protein separation, followed by transfer to a 0.45 µm PVDF membrane (Millipore, Burlington, MA, USA). The membrane was blocked with 5% non-fat dry milk in TBST (Tris-buffered saline with Tween-20) for 1 h at room temperature and then probed with primary antibodies specific to the target proteins overnight at 4 °C. Following TBST washes, the membrane was exposed to the appropriate HRP-conjugated secondary antibody for 2 h at room temperature. Protein bands were visualized using an enhanced chemiluminescence detection system (Pierce Biotech, Rockford, IL, USA) and imaged with the Chemi Doc Imaging System. The primary antibodies utilized were as follows: SHCBP1 (1:200, Cat No. 68647-1-Ig, Proteintech, Rosemont, IL, USA); β-actin (1:20,000, Cat No. 66009-1-Ig, Proteintech, Rosemont, IL, USA); GAPDH (1:50,000, Cat No. 60004-1-Ig, Proteintech, Rosemont, IL, USA). All Western blot experiments were performed in triplicate to ensure reproducibility of the results. The blots shown are representative images from one of the replicates.

### 2.7. Immunohistochemistry (IHC) Staining

Tissue sections (6 µm) fixed in formalin and embedded in paraffin were deparaffinized, rehydrated, and underwent antigen retrieval in citrate buffer (pH 6.0) using microwave heating. Following blocking with 5% BSA, sections were exposed to primary antibodies overnight at 4 °C, then treated with HRP-conjugated secondary antibodies. Immunostaining was visualized using a DAB kit (Beyotime, Shanghai, China), counterstained with hematoxylin, and mounted for examination under a light microscope. The final IHC scores were determined based on the intensity of immunostaining and the percentage of positive cells. Specifically, scores ranging from 0 to 7 were considered indicative of low expression, while scores from 8 to 12 were categorized as high expression. Additionally, the staining procedure and the interpretation of results were independently evaluated by two pathologists to ensure accuracy and consistency.

### 2.8. Co-Expression and Functional Enrichment Analyses

SHCBP1-associated genes in COAD were identified using the LinkedOmics (http://www.linkedomics.org) database. Genes positively or negatively correlated with SHCBP1 expression were ranked according to the correlation coefficient and statistical significance. The top co-expressed genes were visualized as heatmaps and volcano plots. These gene sets were then subjected to GO and KEGG enrichment analyses to explore biological processes and pathways potentially associated with SHCBP1.

### 2.9. Molecular Docking Analysis

Potential SHCBP1-associated proteins were identified using the GeneMANIA (https://genemania.org) and STRING (https://cn.string-db.org) databases. Overlapping candidates from these platforms were selected for further structural exploration. Molecular docking analysis was conducted using the predicted structures of SHCBP1, SHC1, PCLAF, RACGAP1, and KIF23 generated by AlphaFold (https://alphafold.ebi.ac.uk/, accessed on 13 May 2026). Protein preparation, which included the removal of water molecules and addition of polar hydrogens, was performed using AutoDockTools-1.5.7 (MGLTools, The Scripps Research Institute, La Jolla, CA, USA) [24]. Protein-protein docking was then carried out via the Docking Web Server (http://hdock.phys.hust.edu.cn/) [25,26], and the resulting interactions were visualized with PyMOL 2.5.4 (Schrödinger, LLC, New York, NY, USA). These analyses were used to provide structural hypotheses regarding potential SHCBP1-associated interactions rather than direct evidence of binding.

### 2.10. Cell Counting Kit-8 (CCK-8) Assay

Cell proliferation and viability were assessed quantitatively using the CCK-8 assay. Specifically, cells were seeded at a density of 5000 cells per well in 96-well plates and allowed to adhere for 24 h. The CCK-8 reagent (Beyotime, Shanghai, China) was then introduced into each well at a final concentration of 10%. After a 3 h incubation at 37 °C, the absorbance at 450 nm was recorded using a Bio-Rad microplate reader (Bio-Rad Laboratories, Inc., Hercules, CA, USA). The optical density (OD) directly correlated with cell viability, and the proliferation rate was determined by comparing OD values at distinct time points.

### 2.11. EdU Assay

Cells were seeded in a 12-well plate obtained with an EDU kit (Beyotime, Shanghai, China) and then mixed with the 2× EDU working solution in medium, followed by incubation in a humidified incubator at 37 °C under 5% CO_2_. Afterward, the cells were rinsed thrice and fixed using 4% paraformaldehyde, then washed again thrice with a permeabilization solution (Beyotime, Shanghai, China). The reaction solution was made as per the manufacturer’s guidelines and applied to the cells. Subsequently, the cells were stained with the DAPI solution and observed under the fluorescence microscope (Zeiss, Oberkochen, Germany) or flow cytometry (Beckman Coulter, Mumbai, MA, USA).

### 2.12. Colony Formation Assay

Cells were seeded at a low density of 2000 cells/mL in 6-well culture plates and cultured in complete medium for 14 days. Following this, the cells were fixed with 4% paraformaldehyde for 10 min and stained with 0.1% crystal violet (Beyotime, Shanghai, China) for 15 min. Subsequently, individual colonies consisting of at least 50 cells were counted manually.

### 2.13. Subcutaneous Tumor Model

Five-week-old female BALB/c nude mice were purchased from GemPharmatech Co., Ltd. (Nanjing, China). After arrival, animals were acclimatized for 7 days in an SPF barrier facility (12 h light/dark cycle, 22 ± 2 °C, 50–60% humidity) with ad libitum access to sterilized food and water. The experimental unit was an individual animal. Subcutaneous xenografts were established by injecting 3 × 10^5^ HCT116 cells (sh-NC or sh-SHCBP1) in 0.1 mL PBS into the right axilla region. Mice were randomly allocated to two groups (sh-NC and sh-SHCBP1), with 5 mice in each group. No formal a priori sample size calculation was performed; the sample size was determined based on our previous experience with similar xenograft experiments and consideration of animal welfare. Tumor size was monitored twice weekly using a vernier caliper, and tumor volume V = (L × W2)/2, where L represents the long axis and W represents the short axis of the tumor. The primary outcome measure was tumor volume. Outcome assessment, including tumor measurement, was performed by investigators blinded to group allocation. A study protocol including the research question, key design features, and analysis plan was prepared before the study and registered with the institutional animal ethics committee as part of the animal ethics application. Sixteen days post-implantation, the mice were euthanized, and the tumor tissues were excised for further measurement and analysis. No predefined inclusion or exclusion criteria were established for animals or data points, and no animals, experimental units, or data points were excluded from the analysis. Euthanasia was performed via CO_2_ asphyxiation followed by cervical dislocation. Throughout the experiment, no animals exceeded the established tumor burden limits, and all humane endpoints (including tumor ulceration, weight loss exceeding 20%, and impaired mobility) were strictly enforced. All animal procedures were approved by the Institutional Animal Care and Use Committee of the Affiliated Provincial Hospital of Anhui Medical University (approval No. 2025-N(A)-015, approved on January 2025) and were conducted in accordance with applicable institutional guidelines.

### 2.14. Structure-Based Virtual Screening

The AlphaFold algorithm predicted the 3D structure of SHCBP1, and DoGSite identified potential small-molecule binding sites with druggability scores > 0.8. Based on the selected pocket, a pharmacophore model was generated and applied to the ZINC database to filter candidate compounds [27]. A total of 13,127,550 compounds from the ZINC database were initially considered. After pharmacophore-based filtering, the candidate set was reduced to 25,000 compounds for docking evaluation. Molecular docking was then performed to estimate binding affinity, and compounds with docking scores < −8.5 kcal/mol. were retained for further ranking. In total, 381 compounds met this criterion. The top-ranked candidates were visualized in PyMOL, including amino acid residues within 0.5 nm of the predicted binding site and the electrostatic surface properties of the pocket. This virtual screening workflow was intended as an exploratory, hypothesis-generating analysis to identify putative SHCBP1-binding compounds for future experimental validation rather than to establish validated inhibitors.

### 2.15. Statistical Analysis

Statistical analyses were performed using SPSS 21.0 (IBM Corp., Armonk, NY, USA) and GraphPad Prism 8.0 (GraphPad Software, San Diego, CA, USA). Unless otherwise specified, all in vitro experiments were independently repeated at least three times, and quantitative data are presented as mean ± standard deviation (SD). Paired two-tailed Student’s *t*-tests were used for comparisons between matched COAD tissues and adjacent non-tumor tissues. Unpaired two-tailed Student’s *t*-tests were used for comparisons between two independent groups, including cell-based and xenograft experiments. Comparisons among multiple groups were analyzed using one-way analysis of variance (ANOVA) followed by Tukey’s post hoc test, where appropriate. Associations between categorical variables were analyzed using the chi-square test. Kaplan–Meier survival curves were compared using the log-rank test. For xenograft experiments, longitudinal tumor growth curves were analyzed using two-way repeated-measures ANOVA with Geisser–Greenhouse correction to account for within-animal correlations over time. Where appropriate, multiple comparisons between groups at individual time points were performed using Sidak’s post hoc test. A two-sided *p* value < 0.05 was considered statistically significant. Detailed *p* values for the main statistical comparisons are provided in Supplementary Table S3.

## 3. Results

### 3.1. SHCBP1 Expression and Clinical Significance in COAD

**Figure 1:**
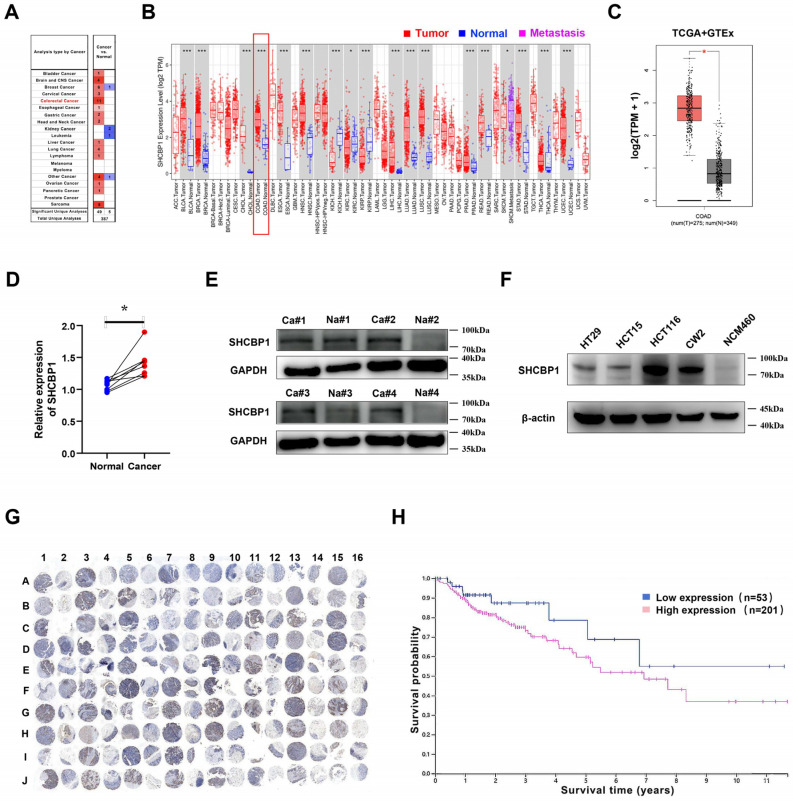
SHCBP1 Expression and Clinical Significance in COAD. (**A**,**B**) Oncomine and TIMER databases analyses of *SHCBP1* mRNA expression across different cancer types (Red and blue indicate upregulated and downregulated expression, respectively. Red font and boxes highlight COAD-related results. * *p* < 0.05; *** *p* < 0.001). (**C**) Analysis of SHCBP1 mRNA levels in COAD tumors (*n* = 275) and normal tissues (*n* = 349) using TCGA and GTEx data. (**D**) RT-qPCR analysis of *SHCBP1* mRNA levels in COAD tumors compared to adjacent normal tissues. (**E**) Western blot analysis of SHCBP1 protein expression in COAD tumors compared to adjacent normal tissues. (**F**) Western blot comparison of SHCBP1 protein levels in COAD cell lines (HT29, HCT15, HCT116, CW2) and normal colon mucosal cell line NCM460. (**G**) IHC staining of SHCBP1 protein expression in COAD tissues microarray (Odd-numbered columns represent cancerous tissue, and even-numbered columns represent adjacent tissue). (**H**) Kaplan–Meier analysis of overall survival in COAD patients with high (*n* = 201) versus low SHCBP1 (*n* = 53) expression levels based on HPA database. Quantitative analyses corresponding to panels (**E**–**G**) are provided in Supplementary Figure S1. The uncropped blots are shown in Supplementary Materials.

The expression levels of *SHCBP1* across various cancers were examined through the Oncomine and TIMER databases, and the analysis showed that *SHCBP1* levels were significantly higher in tumor tissues than in normal tissues, particularly in COAD (Figure 1A,B). These findings were further validated using the GEPIA2 database (Figure 1C). RT-qPCR and Western blot analyses further confirmed that SHCBP1 expression was elevated in COAD tissues compared with adjacent normal tissues (Figure 1D,E), and the corresponding quantitative analysis is shown in Supplementary Figure S1A. Consistently, SHCBP1 expression was higher in COAD cell lines (HT29, HCT15, HCT116, and CW2) than in the normal colonic epithelial cell line NCM460 (Figure 1F), with quantitative analysis provided in Supplementary Figure S1B. In addition, IHC staining performed on a tissue microarray containing 80 paired COAD and adjacent normal tissues showed stronger SHCBP1 staining in tumor tissues (Figure 1G), and semi-quantitative analysis further supported this difference (Supplementary Figure S1C). The Human Protein Atlas database revealed a correlation between high SHCBP1 expression and poor prognosis in COAD patients (Figure 1H). Together, these results indicate that SHCBP1 is upregulated in COAD and is associated with unfavorable survival outcomes in publicly available datasets.

### 3.2. SHCBP1 Methylation Status in COAD

**Figure 2:**
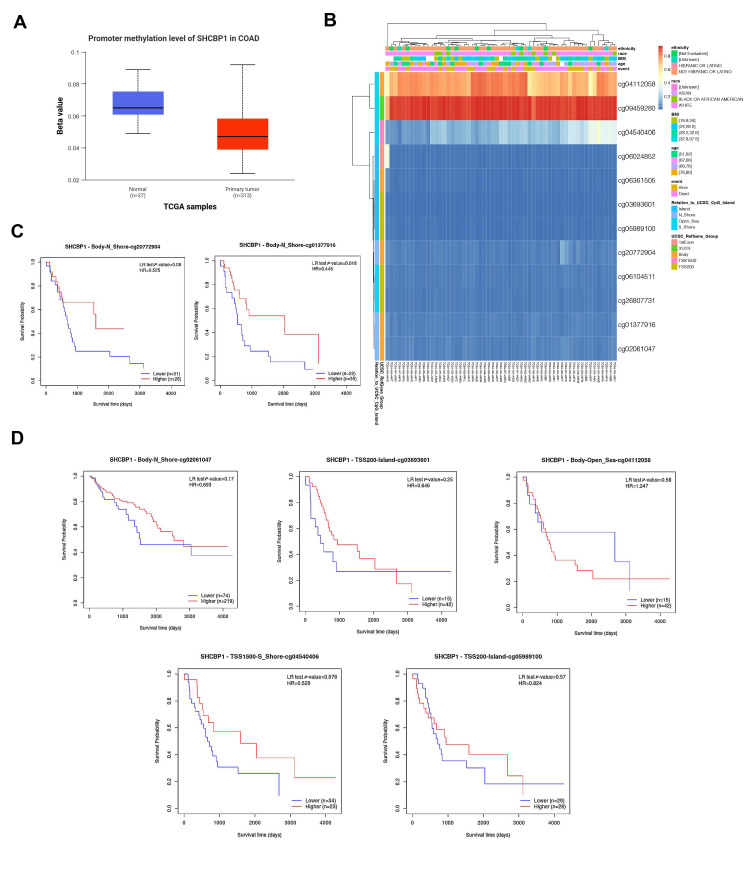
*SHCBP1* Methylation Status in COAD. (**A**) Comparison of *SHCBP1* promoter methylation levels in COAD samples from the TCGA dataset. (**B**) Methylation profiles of *SHCBP1* CpG sites across COAD samples. (**C**) Association between methylation levels at specific *SHCBP1* CpG sites (e.g., cg20772904 and cg01377916) and overall survival in COAD patients. (**D**) Survival analysis for other *SHCBP1* CpG sites (e.g., cg02061047, cg03693601, cg04112058, cg04540406, and cg05989100).

Aberrant DNA methylation, particularly in gene promoter regions, is a well-established mechanism of gene regulation implicated in tumorigenesis [22]. We analyzed *SHCBP1* DNA methylation and its prognostic implications using the UALCAN database. Tumor tissues showed significantly lower DNA methylation levels than adjacent normal tissues, suggesting that elevated SHCBP1 expression in COAD may be linked to reduced promoter methylation (Figure 2A). Investigation of individual CpG sites in the *SHCBP1* gene showed most sites were hypomethylated except for two (cg04112058 and cg09459280) with increased methylation in tumor samples (Figure 2B). Hypomethylation at cg20772904 and cg01377916 was significantly associated with poorer survival in COAD patients (*p* < 0.05) (Figure 2C). Although some sites did not reach statistical significance, a trend towards a negative correlation between low methylation and poorer prognosis was observed (Figure 2D). These findings suggest that SHCBP1 hypomethylation may be associated with its elevated expression and unfavorable prognosis in COAD.

### 3.3. Co-Expression and Enrichment Analysis of SHCBP1 in COAD

**Figure 3:**
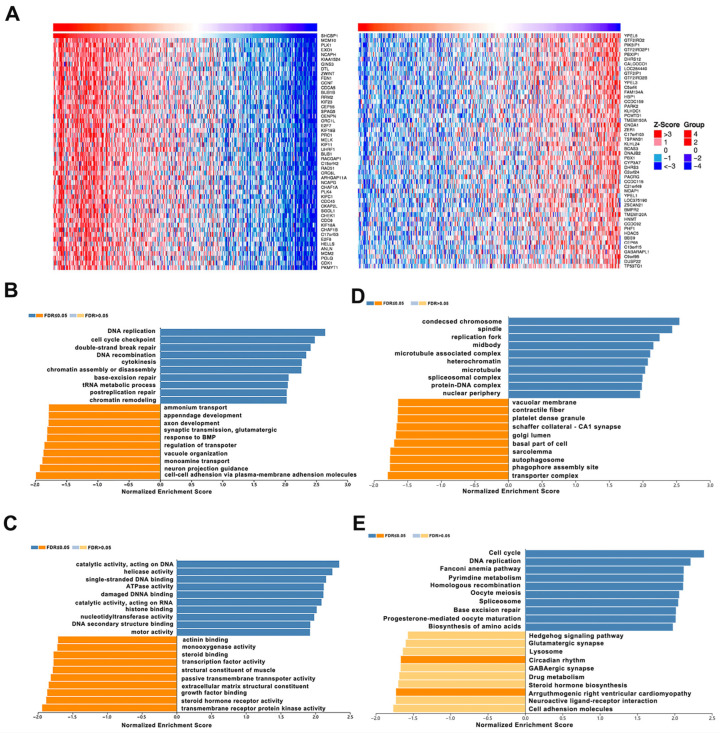
Co-expressed profiles and enriched analyses of SHCBP1 in COAD (**A**) The heatmap of the top 50 genes significantly positively or negatively correlated with SHCBP1 expression. (**B**) Biological process enrichment analysis for SHCBP1 co-expressed genes. (**C**) Molecular functions enrichment analysis for SHCBP1 co-expressed genes. (**D**) Cellular component enrichment analysis for SHCBP1 co-expressed genes. (**E**) KEGG pathway enrichment analysis for SHCBP1 co-expressed genes.

To better understand the biological function of SHCBP1 in COAD, we performed a co-expression analysis utilizing the LinkedOmics database. Our analysis revealed 7984 genes that exhibited a notable positive correlation with the expression of *SHCBP1*, while 11,844 genes exhibited a significant negative correlation. The top 50 correlated genes strongly correlated with SHCBP1 are shown in the heatmap (Figure 3A). Next, enrichment analysis was performed based on GO functions and KEGG pathways using GSEA methods. The main enriched biological processes included “DNA replication” and “cell cycle checkpoint” (Figure 3B), while the most enriched molecular functions were “catalytic activity, acting on DNA” and “helicase activity” (Figure 3C). The predominant terms for cellular components were “condensed chromosome” and “spindle” (Figure 3D). Analysis of KEGG pathways highlighted “Cell cycle” and “DNA replication” as the most enriched pathways (Figure 3E). These findings suggest that SHCBP1 is associated with gene networks related to cell-cycle regulation and DNA replication in COAD.

### 3.4. Exploratory In Silico Analysis of Predicted SHCBP1-Associated Interaction Networks

**Figure 4:**
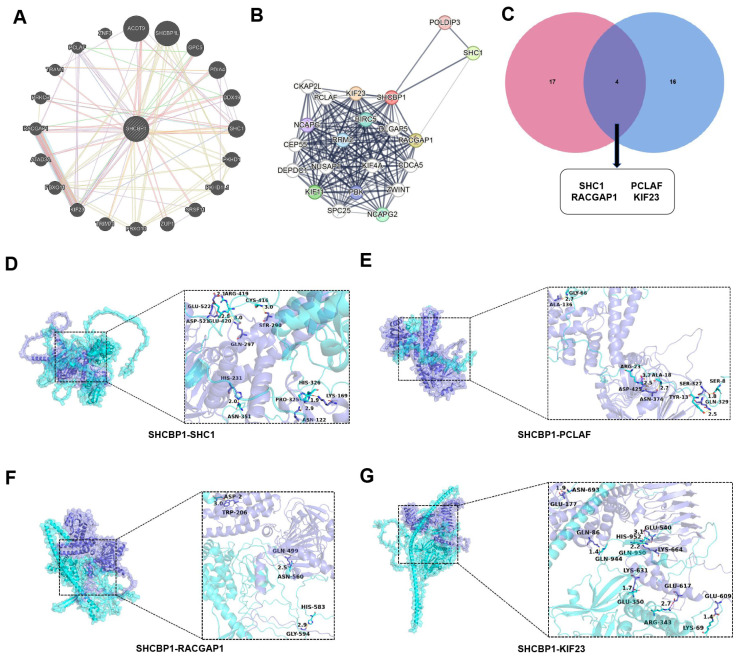
Exploratory in silico analysis of predicted SHCBP1-associated interaction networks and structural docking models. (**A**,**B**) Interaction network of SHCBP1 constructed by GeneMANIA and STRING. (**C**) Venn diagram showing the overlap of SHCBP1-interacting proteins identified from different datasets. (**D**–**G**) Molecular docking and structural interaction analysis of SHCBP1 with its key interacting proteins: SHC1 (**D**), PCLAF (**E**), RACGAP1 (**F**), and KIF23 (**G**).

As part of the exploratory computational component of this study, we examined proteins potentially associated with SHCBP1 using the GeneMANIA and STRING databases (Figure 4A,B). These analyses identified SHC1, PCLAF, RACGAP1, and KIF23 as candidate SHCBP1-associated proteins (Figure 4C). To further explore the structural plausibility of these predicted associations, protein-protein docking analyses were performed using AlphaFold-predicted structures of SHCBP1 and the candidate proteins. The docking models suggested structurally compatible interaction interfaces between SHCBP1 and SHC1 (Figure 4D), PCLAF (Figure 4E), RACGAP1 (Figure 4F), and KIF23 (Figure 4G). These findings should be interpreted as in silico, hypothesis-generating observations rather than experimentally validated evidence of direct protein interaction.

### 3.5. Effects of SHCBP1 Knockdown on COAD Cell Proliferation and Tumor Growth

**Figure 5:**
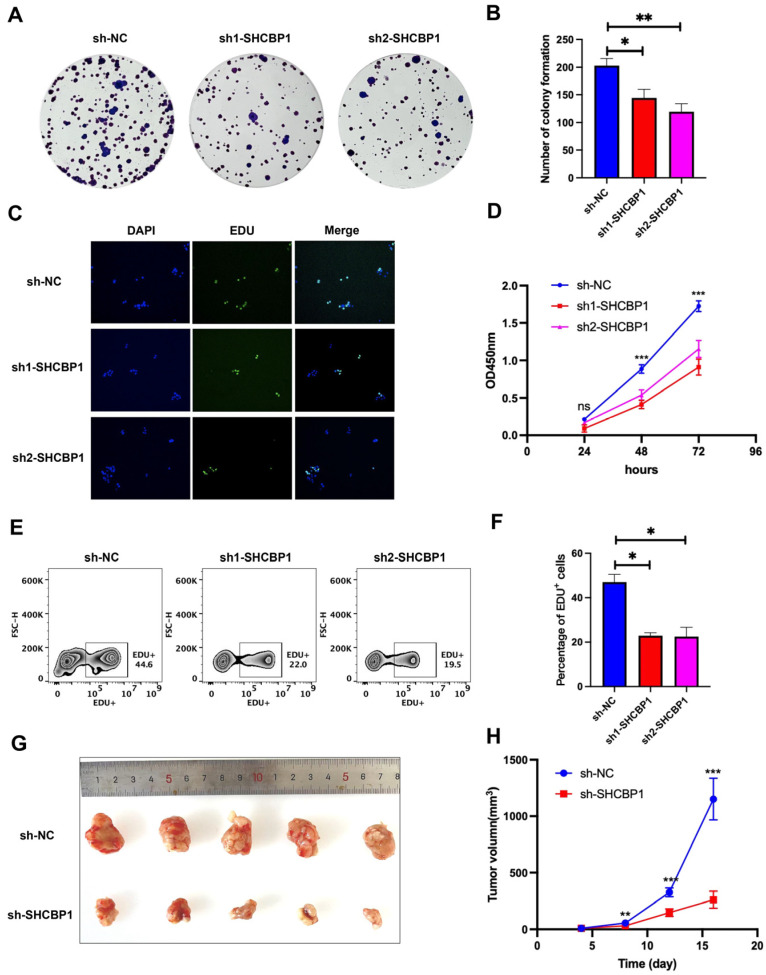
Effects of SHCBP1 knockdown on cell proliferation and tumor growth in COAD. (**A**) Representative images of colony formation assays showing reduced colony formation in SHCBP1 knockdown groups and negative control. (**B**) Quantitative analysis of colony numbers from the colony formation assay. (**C**) EdU staining images of cells in SHCBP1 knockdown groups and control group (Original magnification, ×200). (**D**) Cell proliferation curves generated from CCK-8 assays. (**E**) Flow cytometry analysis of EdU-positive cells. (**F**) Quantification of EdU-positive cells from flow cytometry analysis. (**G**) Representative images of tumors excised from subcutaneous tumor xenograft models. (**H**) Tumor growth curves in subcutaneous tumor xenograft models (Tumor growth curves were analyzed using two-way repeated-measures ANOVA with Geisser-Greenhouse correction, followed by Sidak’s multiple-comparisons test. Significant differences between groups were observed at days 8, 12, and 16). (ns, not significant, * *p* < 0.05, ** *p* < 0.01, *** *p* < 0.001).

Based on our enrichment analysis results, SHCBP1 may be associated with the “DNA replication” and “Cell cycle” signaling pathways, indicating its potential contribution to cell proliferation and tumor growth. Effective SHCBP1 knockdown in HCT116 cells was confirmed by RT-qPCR and Western blot analyses (Supplementary Figure S2A–D). To further clarify this phenomenon, we conducted a series of in vitro and in vivo experiments. Colony formation assays indicated that reducing SHCBP1 expression in HCT116 cells markedly diminished clonogenic capacity relative to control cells (Figure 5A,B). EdU assays revealed a substantial decrease in proliferative activity in cells with diminished SHCBP1 expression (Figure 5C). CCK-8 assays further demonstrated a pronounced reduction in cell viability following SHCBP1 downregulation (Figure 5D). Flow cytometry analysis of EdU-labeled HCT116 cells showed a marked decrease in the proportion of EdU-positive cells after SHCBP1 attenuation (Figure 5E,F). Within a subcutaneous tumor xenograft model, mice with reduced SHCBP1 expression exhibited significantly inhibited tumor growth (Figure 5G,H). Together, these results support a role for SHCBP1 in promoting the proliferative capacity and tumor growth of COAD cells in the HCT116 model.

### 3.6. Computational Drug Target Exploration: Structure-Based Virtual Screening as a Hypothesis-Generating Analysis

**Figure 6:**
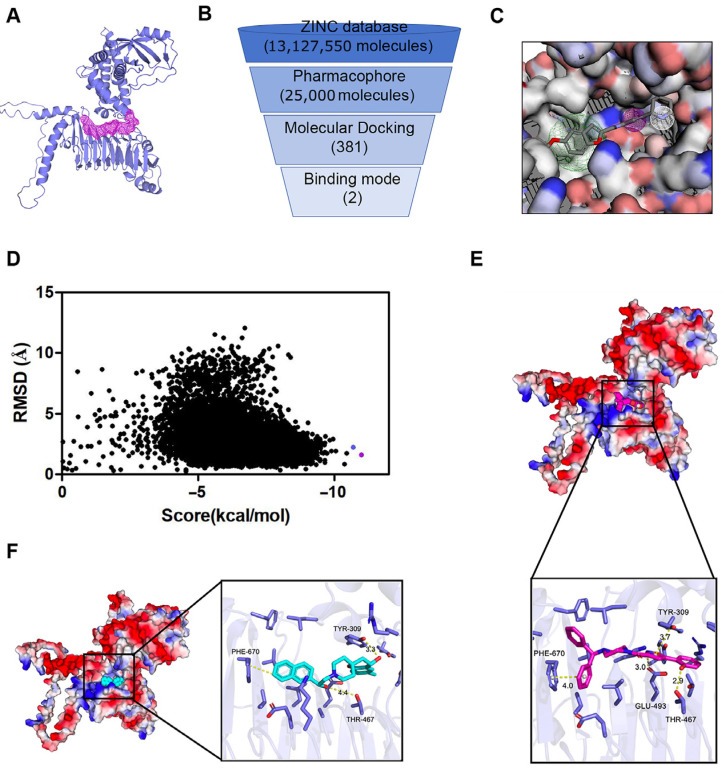
Structure-based virtual screening of putative SHCBP1-binding compounds. (**A**) Cartoon representation of the Alphafold predicted structure of SHCBP1 protein, where the magenta grid is the predicted drug target site; (**B**) Virtual screening flow chart. (**C**) Pharmacophore modeling results based on protein pockets. (**D**) Docking free energy scores of the top 25,000 molecules after pharmacophore screening. (**E**,**F**) Binding mode diagrams of the first and second molecules in virtual screening scores.

Given the biological relevance of SHCBP1 in COAD, we next performed an exploratory structure-based virtual screening workflow as part of the computational component of this study to prioritize compounds with predicted binding potential. The three-dimensional structure of SHCBP1 was predicted to identify a potential drug-binding site (Figure 6A). We then conducted computer-based virtual screening from the ZINC database (Figure 6B illustrates the screening process). Using a pharmacophore model of the predicted binding site (Figure 6C), we efficiently narrowed the candidate compounds from 13,127,550 to 25,000. Subsequent molecular docking of these 25,000 compounds revealed the distribution of docking scores and root mean square deviations (RMSD) value (Figure 6D). This analysis identified 381 compounds with docking scores below −8.5 kcal/mol. Among these, ZINC00020137678 and ZINC00012293622 showed favorable predicted docking scores within the selected SHCBP1 pocket, as indicated by their exceptionally low docking scores of −10.985 and −10.676, respectively. Electrostatic surface maps and close-up stick representations (Figure 6E,F) highlight the plausible interactions between these two compounds and residues within the predicted SHCBP1 pocket. These findings provide preliminary in silico candidates with predicted SHCBP1-binding potential for future biochemical and cellular validation.

## 4. Discussion

Despite advancements in multidisciplinary treatments, including surgery, chemotherapy, and immunotherapy, the prognosis for COAD remains unfavorable, with stagnant overall survival rates [25]. Therefore, identifying novel molecular markers associated with tumor progression may help improve prognostic evaluation and provide a basis for future targeted therapeutic development. SHCBP1 has increasingly been recognized as a cancer-related protein associated with proliferation-related signaling and cell-cycle regulation in several malignancies [5]. However, its precise role and clinical relevance in COAD pathogenesis remain poorly understood.

In the present study, we combined experimentally supported biological analyses with exploratory in silico analyses to investigate the potential role of SHCBP1 in COAD. The main conclusions of the study are supported by expression analyses, tissue-level validation, and functional experiments, whereas the interaction-network and virtual-screening results should be interpreted as hypothesis-generating findings that require further validation. Within this framework, our results showed that SHCBP1 was upregulated in COAD tissues and associated with poorer prognosis, suggesting potential clinical relevance in this disease context. It should be noted that the survival analyses were based primarily on publicly available datasets with limited clinical annotation. Therefore, the observed association between SHCBP1 expression and patient outcome may have been influenced by additional factors such as treatment, mutation status, or disease stage, and does not establish SHCBP1 as an independent prognostic factor. To explore a possible basis for SHCBP1 upregulation, we further examined its methylation status [26]. Our analyses showed that SHCBP1 promoter methylation was reduced in COAD tissues compared with normal tissues, and hypomethylation at specific CpG sites was associated with worse patient survival. These findings suggest that epigenetic dysregulation may contribute, at least in part, to SHCBP1 overexpression in COAD.

Bioinformatic analyses further indicated that SHCBP1-associated gene networks were enriched in “DNA replication,” “Cell cycle” and proliferation-related processes. In addition, interaction-network and docking analyses suggested several proteins that may be functionally associated with SHCBP1. Among them, the interaction between SHCBP1 and SHC1 has been reported previously, whereas the associations with PCLAF, RACGAP1, and KIF23 remain computationally inferred in the present study [27]. Notably, PCLAF, RACGAP1, and KIF23 have all been implicated in cell division and proliferation [28,29,30]. Structural docking analyses supported the plausibility of these predicted associations and provided a preliminary basis for future mechanistic investigation.

Functionally, our in vitro and in vivo results support a role for SHCBP1 in promoting tumor cell proliferation and tumor growth. In HCT116 cells, SHCBP1 knockdown reduced colony formation, EdU incorporation, and cell growth, and also suppressed xenograft tumor growth. These findings are consistent with the enrichment results and support the biological relevance of SHCBP1 in the HCT116 COAD model.

We also performed structure-based virtual screening as an exploratory extension of the biological findings to examine whether SHCBP1 may have tractable binding pockets for future drug-development studies. This analysis was retained to provide a preliminary computational perspective on the targetability of SHCBP1, but it does not constitute experimental evidence of inhibitor activity or direct binding. Using this workflow, we prioritized several candidate compounds, among which ZINC00020137678 and ZINC00012293622 showed favorable predicted docking scores within the selected SHCBP1 pocket. These observations provide initial in silico leads for future biochemical and pharmacological evaluation.

Several limitations of this study should be acknowledged. First, the methylation findings were derived primarily from public databases and were not independently validated in an external patient cohort. These analyses are exploratory in nature and should be interpreted with caution, and the observations do not constitute definitive mechanistic evidence. Second, the SHCBP1-associated proteins predicted through bioinformatic analyses and structural docking, including SHC1, PCLAF, RACGAP1, and KIF23, remain bioinformatically inferred candidates and require direct experimental validation using approaches such as co-immunoprecipitation or related biochemical assays. Third, although enrichment analysis, interaction prediction, and docking analyses suggested possible SHCBP1-related pathways and molecular associations, these findings remain inferential and do not establish direct mechanistic links. Fourth, the functional role of SHCBP1 was examined mainly in a single COAD cell line (HCT116), which may limit the generalizability of the observed phenotypes across genetically diverse COAD models. Finally, the compounds prioritized by virtual screening remain preliminary in silico candidates with predicted SHCBP1-binding potential and have not yet been validated by biochemical binding assays, cell-based functional assays, or toxicity assessments.

## 5. Conclusions

In summary, our study shows that SHCBP1 is upregulated in COAD and is associated with unfavorable prognosis. Promoter hypomethylation may contribute to its increased expression. Integrated bioinformatic analyses suggested that SHCBP1 is associated with cell-cycle and DNA-replication related gene networks, and functional experiments supported its role in promoting proliferative capacity and tumor growth in the HCT116 COAD model. In addition, exploratory computational analyses identified predicted SHCBP1-associated interactions and candidate compounds with predicted binding potential. Overall, SHCBP1 may represent a candidate biomarker and a potentially relevant therapeutic target in COAD that warrants further investigation.

## Data Availability

The data presented in this study are available on request due to privacy concerns.

## Supplementary Materials

The following supporting information can be downloaded at: https://www.mdpi.com/article/10.3390/curroncol33050295/s1, Table S1: Databases for Bioinformatics Analyses with Corresponding URLs; Table S2: shRNA sequences and RT-qPCR primers; Table S3: The *p*-values for statistical tests in this manuscript; Figure S1: Quantitative analyses corresponding to Figure 1E–G; Figure S2: Validation of SHCBP1 knockdown in HCT116 cells.
