# Supplementary material for: SHCBP1 Is Upregulated in Colon Adenocarcinoma and Promotes Tumor Cell Proliferation and Growth
Source: Curr Oncol. 2026 May 19;33(5):295. doi: 10.3390/curroncol33050295 (PMC13206487; doi:10.3390/curroncol33050295)
Supplement: Supplementary file 1 [file curroncol-33-00295-s001.zip › curroncol-4211370-original-images.pdf]

Uncropped Gels and Blots

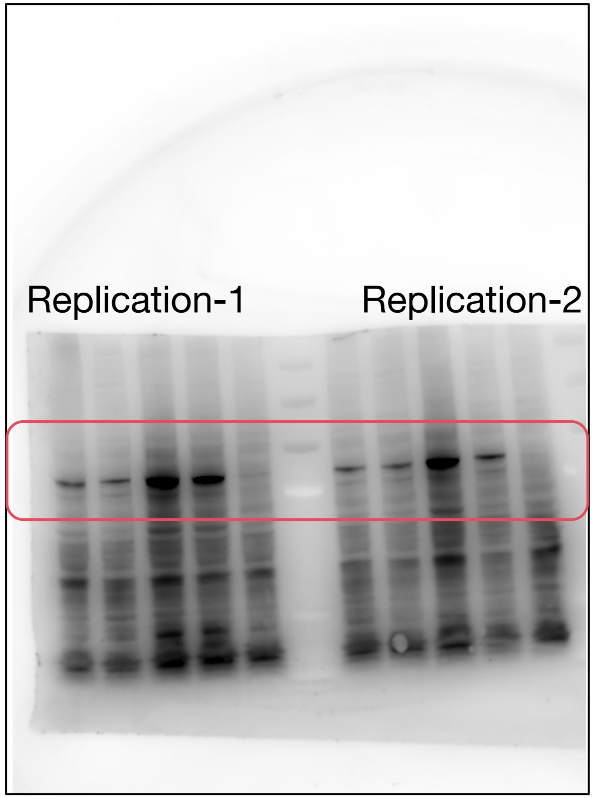

This Gel (left) was used in the Fig1G

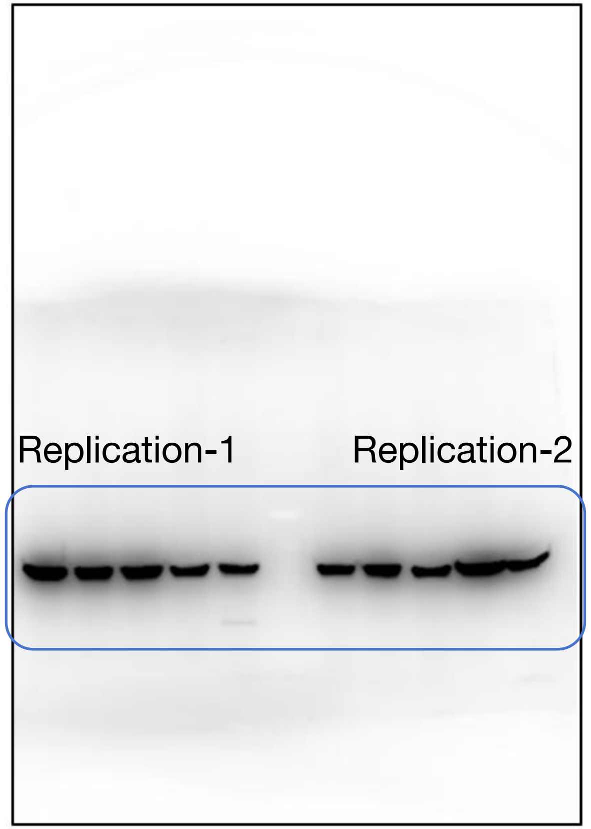

This Gel (left) was used in the Fig1G

Antibody information:
SHCBP1 Polyclonal antibody
Cat No. 66009-1-Ig （Proteintech）
Observed molecular weight： 75 kDa

Antibody information:
Beta Actin Monoclonal antibody
Cat No. 66009-1-Ig （Proteintech）
Observed molecular weight： 42 kDa

Replication-3

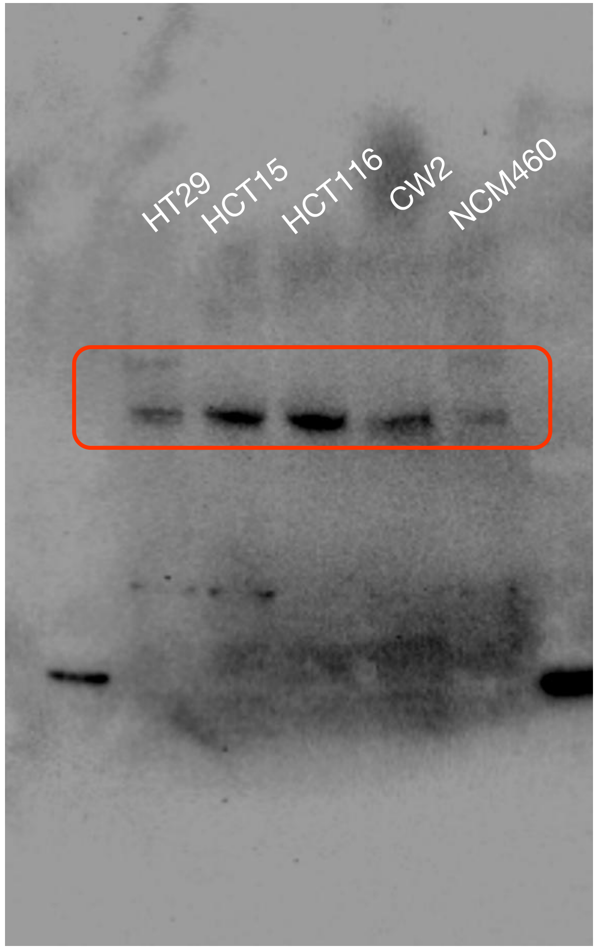

Replication-3

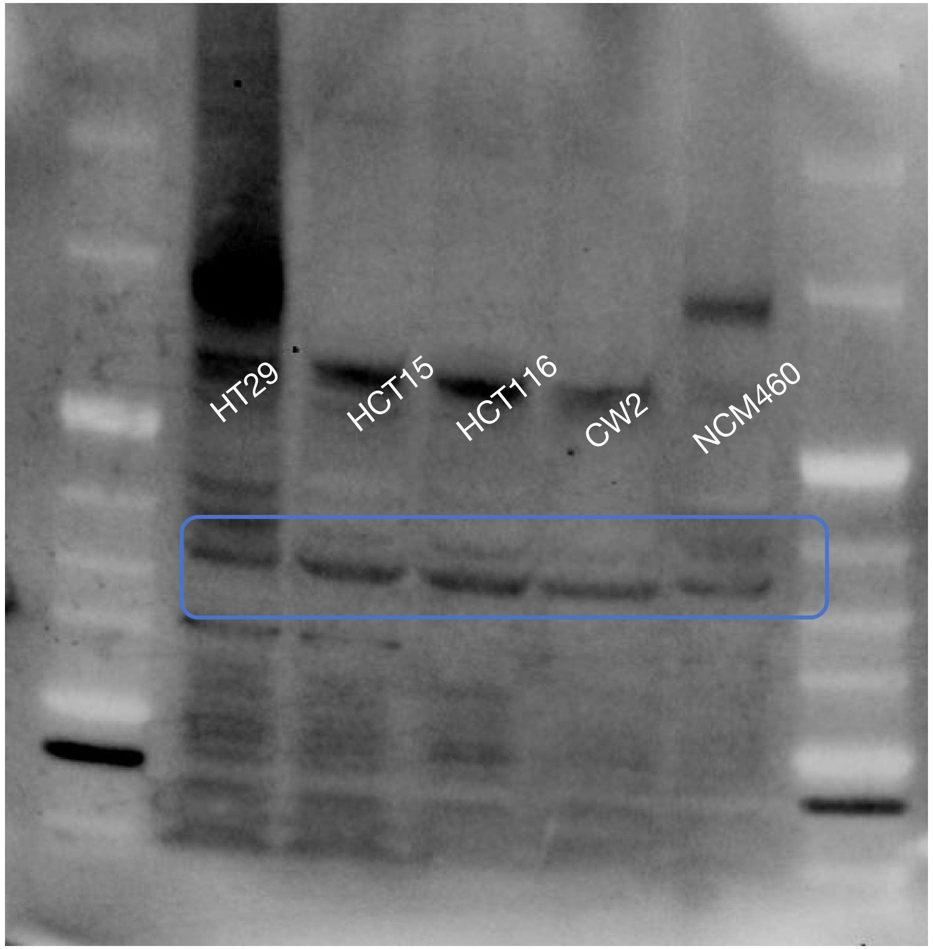

Antibody information:

SHCBP1 Polyclonal antibody

Cat No. 66009-1-Ig （Proteintech）

Observed molecular weight： 75 kDa

Antibody information:

Beta Actin Monoclonal antibody

Cat No. 66009-1-Ig （Proteintech）

Observed molecular weight： 42 kDa

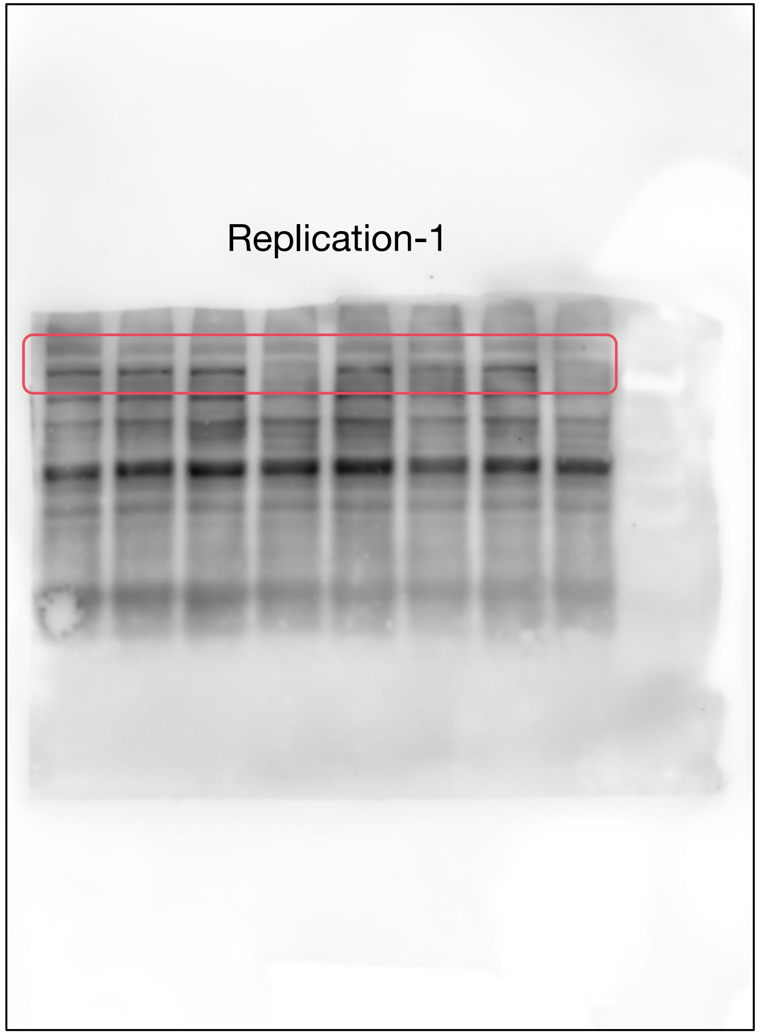

This Gel was used in the Fig1F

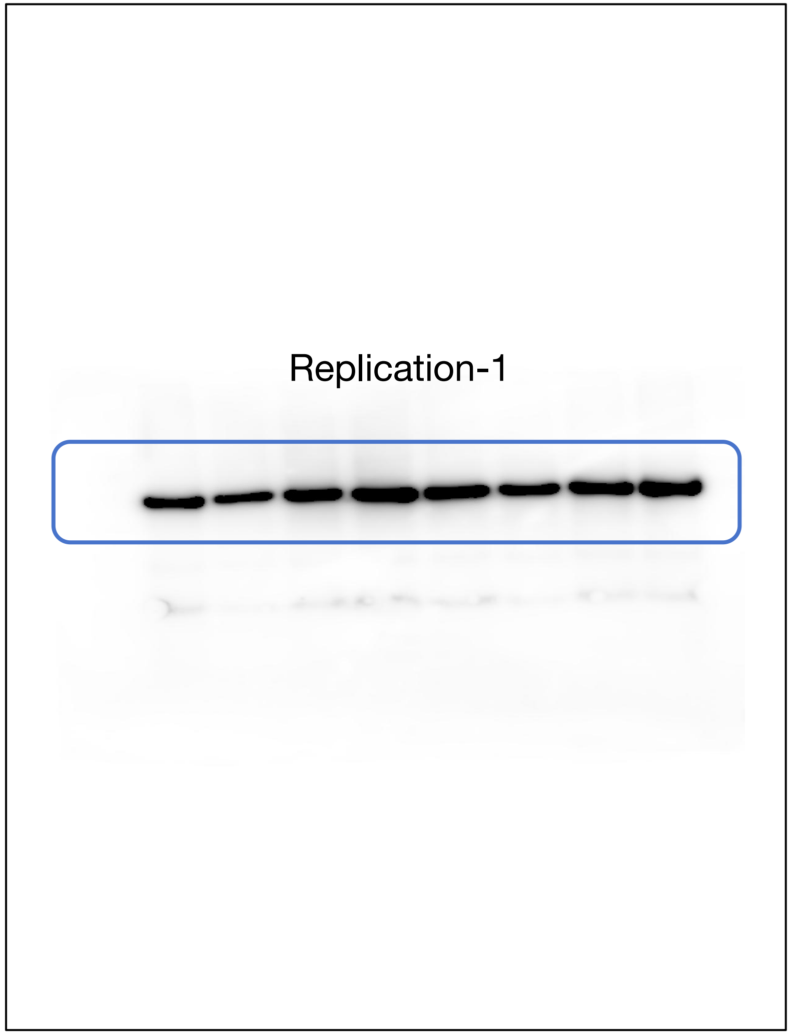

This Gel (left) was used in the Fig1F

Antibody information:
SHCBP1 Polyclonal antibody
Cat No. 66009-1-Ig （Proteintech）
Observed molecular weight： 75 kDa

Antibody information:
GAPDH Monoclonal antibody
Cat No. 60004-1-Ig （Proteintech）
Observed molecular weight： 36 kDa

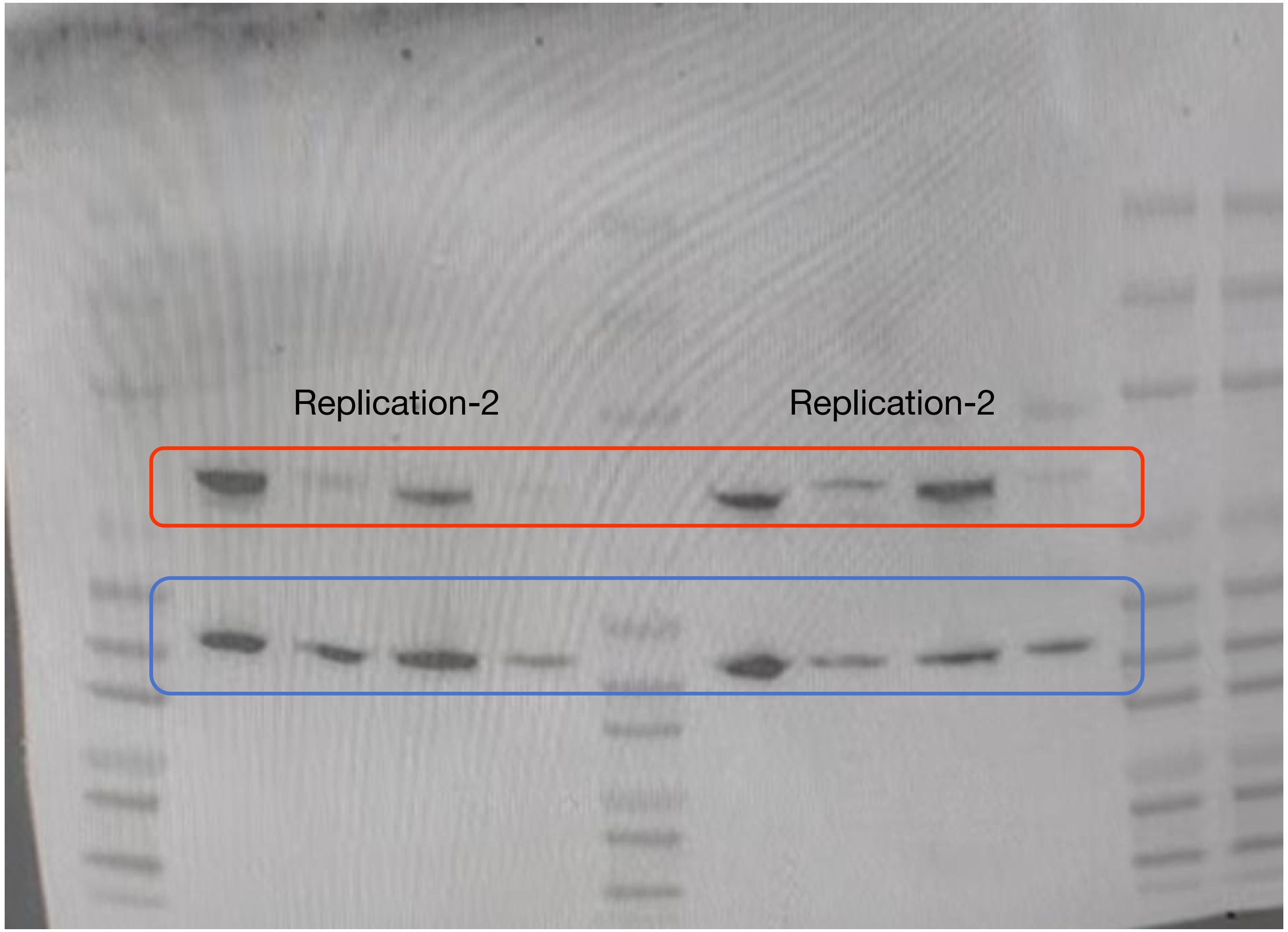

Antibody information:
SHCBP1 Polyclonal antibody
Cat No. 66009-1-Ig （Proteintech）
Observed molecular weight： 75 kDa

Antibody information:
Beta Actin Monoclonal antibody
Cat No. 66009-1-Ig （Proteintech）
Observed molecular weight： 42 kDa

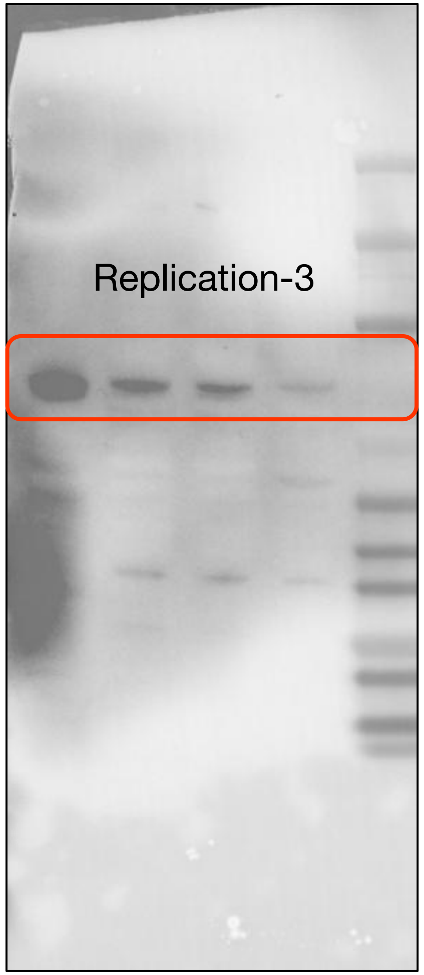

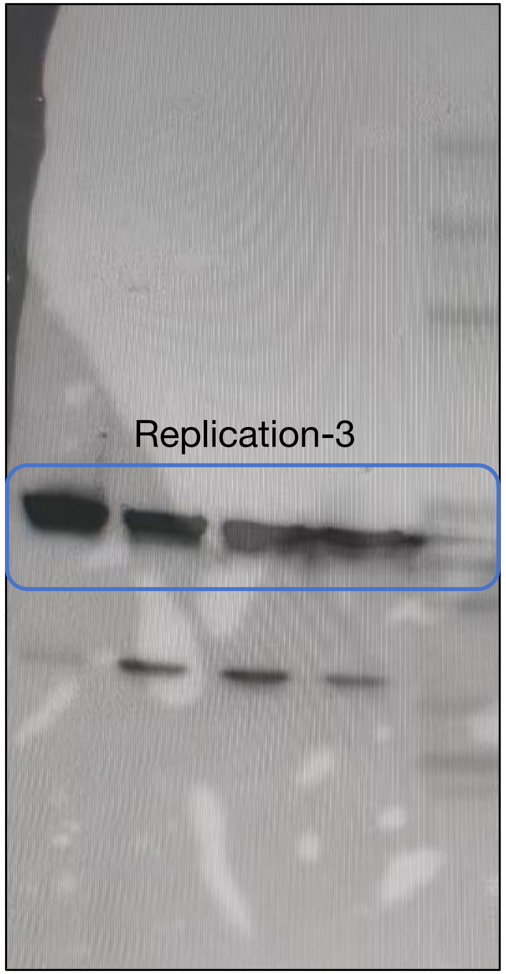

Antibody information:
SHCBP1 Polyclonal antibody
Cat No. 66009-1-Ig（Proteintech）
Observed molecular weight：75 kDa

Antibody information:
Beta Actin Monoclonal antibody
Cat No. 66009-1-Ig（Proteintech）
Observed molecular weight：42 kDa

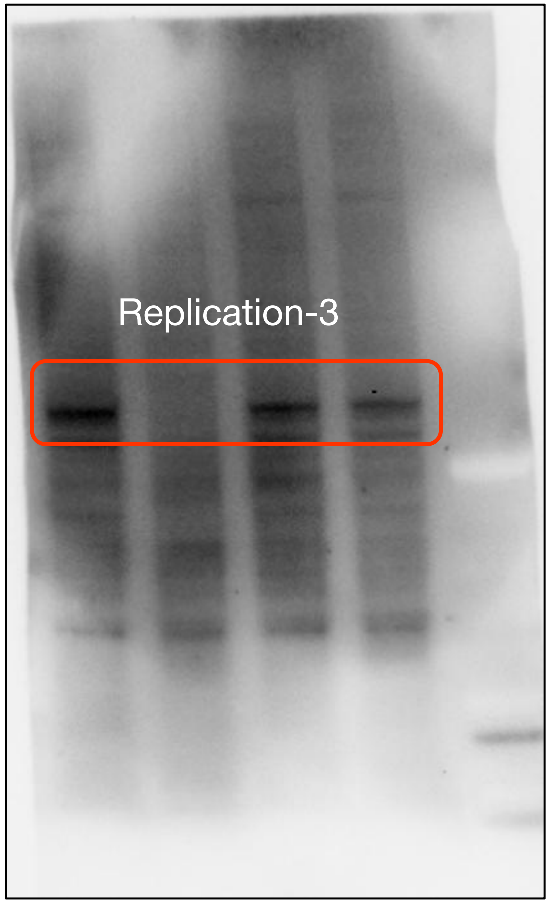

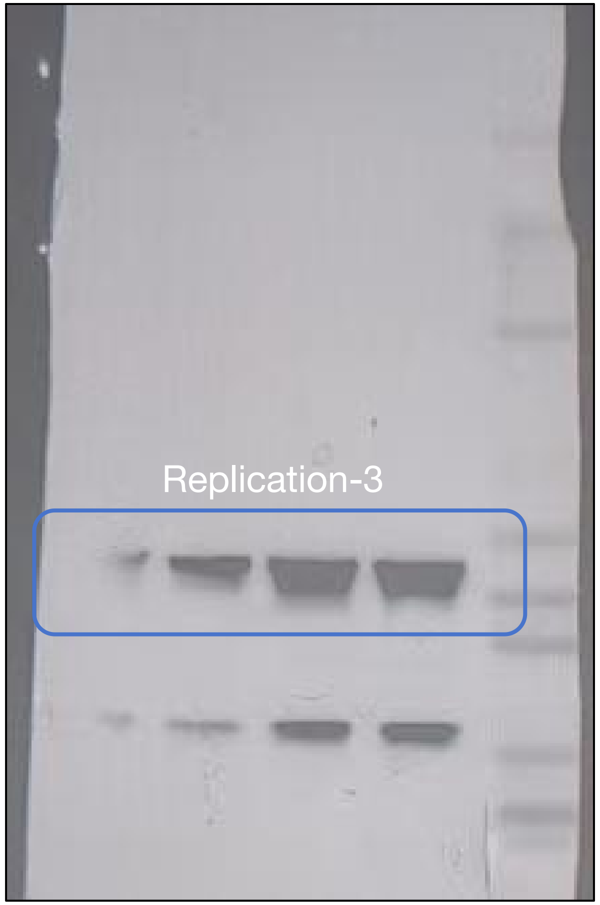

Antibody information:
SHCBP1 Polyclonal antibody
Cat No. 66009-1-Ig （Proteintech）
Observed molecular weight： 75 kDa

Antibody information:
Beta Actin Monoclonal antibody
Cat No. 66009-1-Ig （Proteintech）
Observed molecular weight： 42 kDa

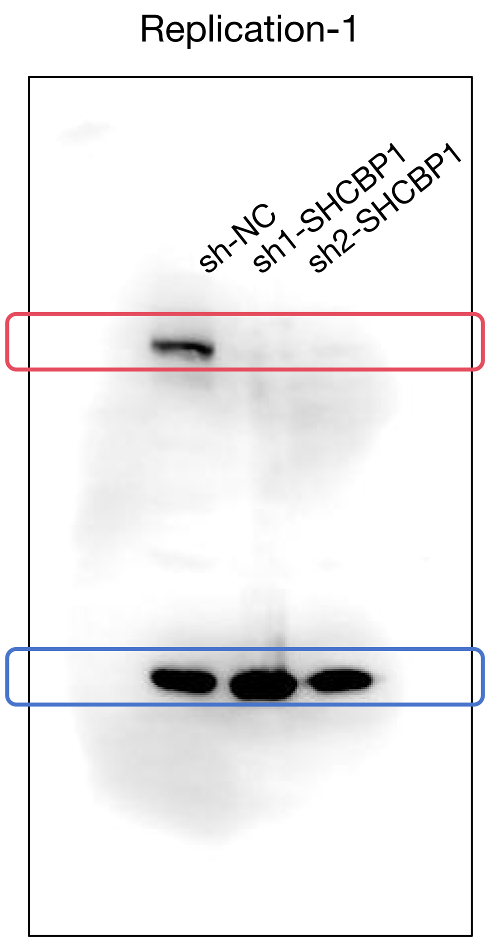

Antibody information:
SHCBP1 Polyclonal antibody
Cat No. 66009-1-Ig （Proteintech）
Observed molecular weight： 75 kDa

Antibody information:
GAPDH Monoclonal antibody
Cat No. 60004-1-Ig （Proteintech）
Observed molecular weight： 36 kDa

Replication-2

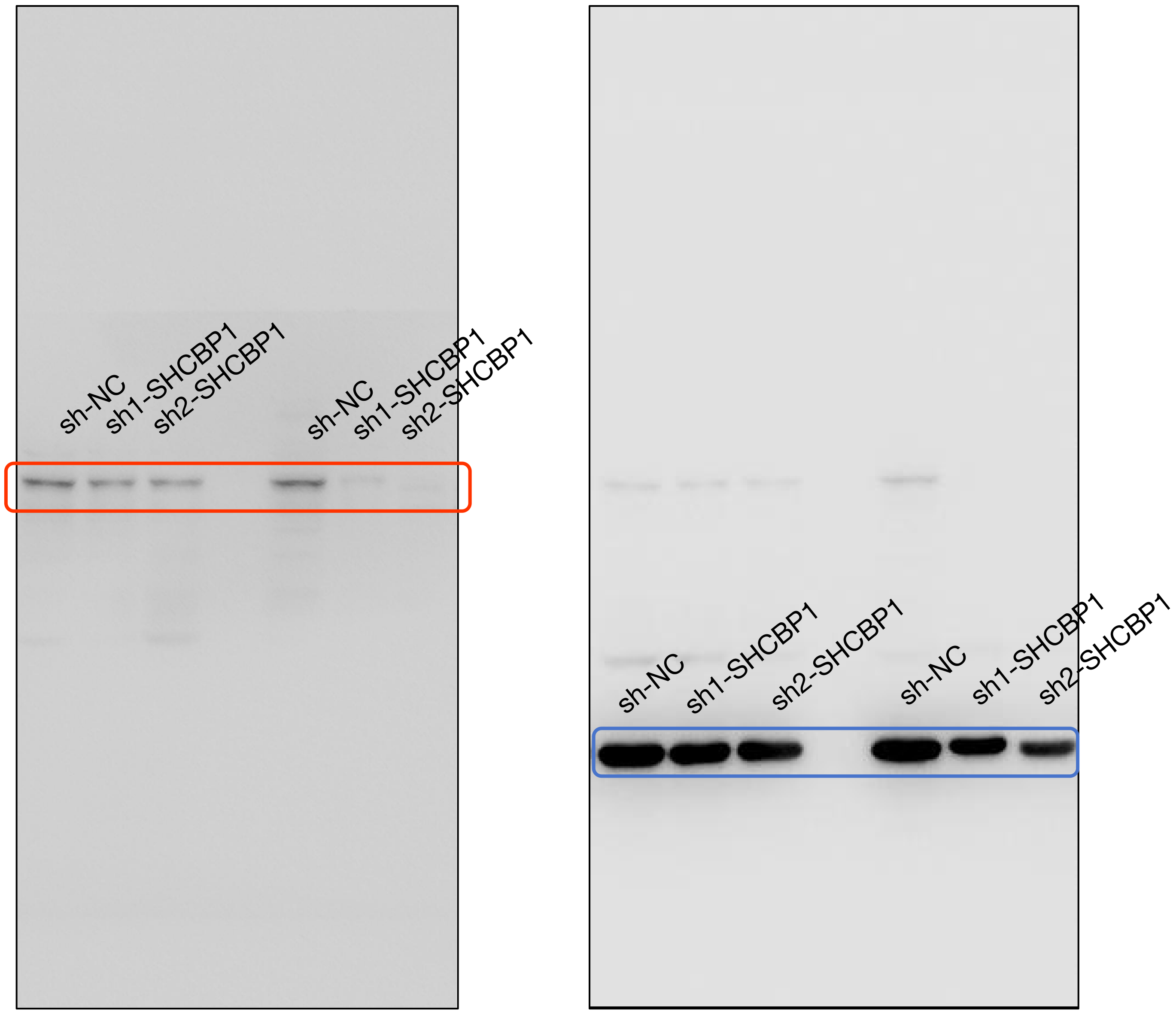

Antibody information:
SHCBP1 Polyclonal antibody
Cat No. 66009-1-Ig （Proteintech）
Observed molecular weight： 75 kDa

Antibody information:
GAPDH Monoclonal antibody
Cat No. 60004-1-Ig （Proteintech）
Observed molecular weight： 36 kDa

Replication-3

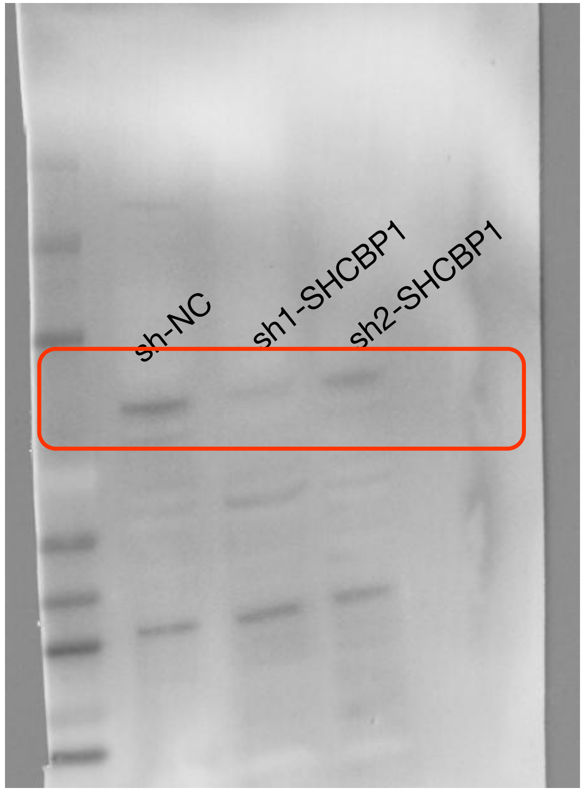

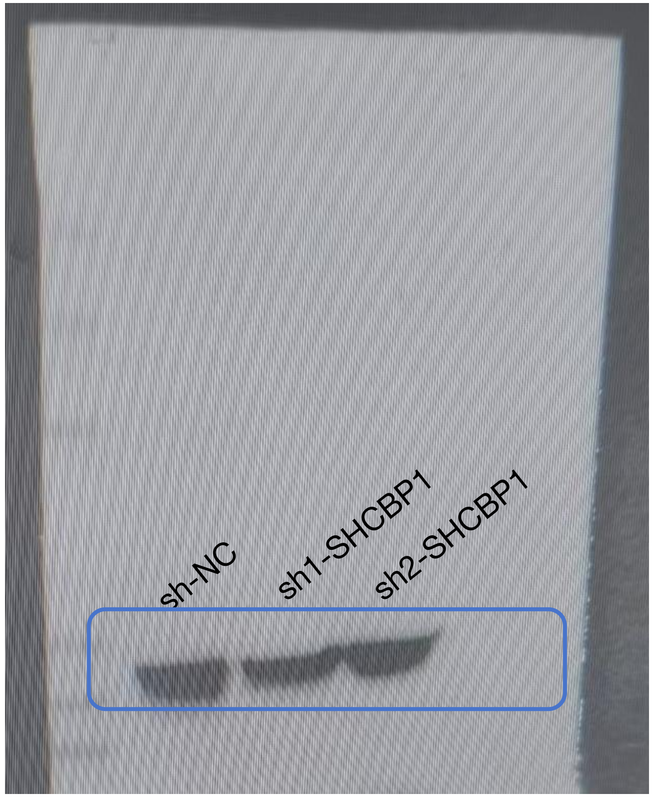

Antibody information:
SHCBP1 Polyclonal antibody
Cat No. 66009-1-Ig （Proteintech）
Observed molecular weight： 75 kDa

Antibody information:
Beta Actin Monoclonal antibody
Cat No. 66009-1-Ig （Proteintech）
Observed molecular weight： 42 kDa

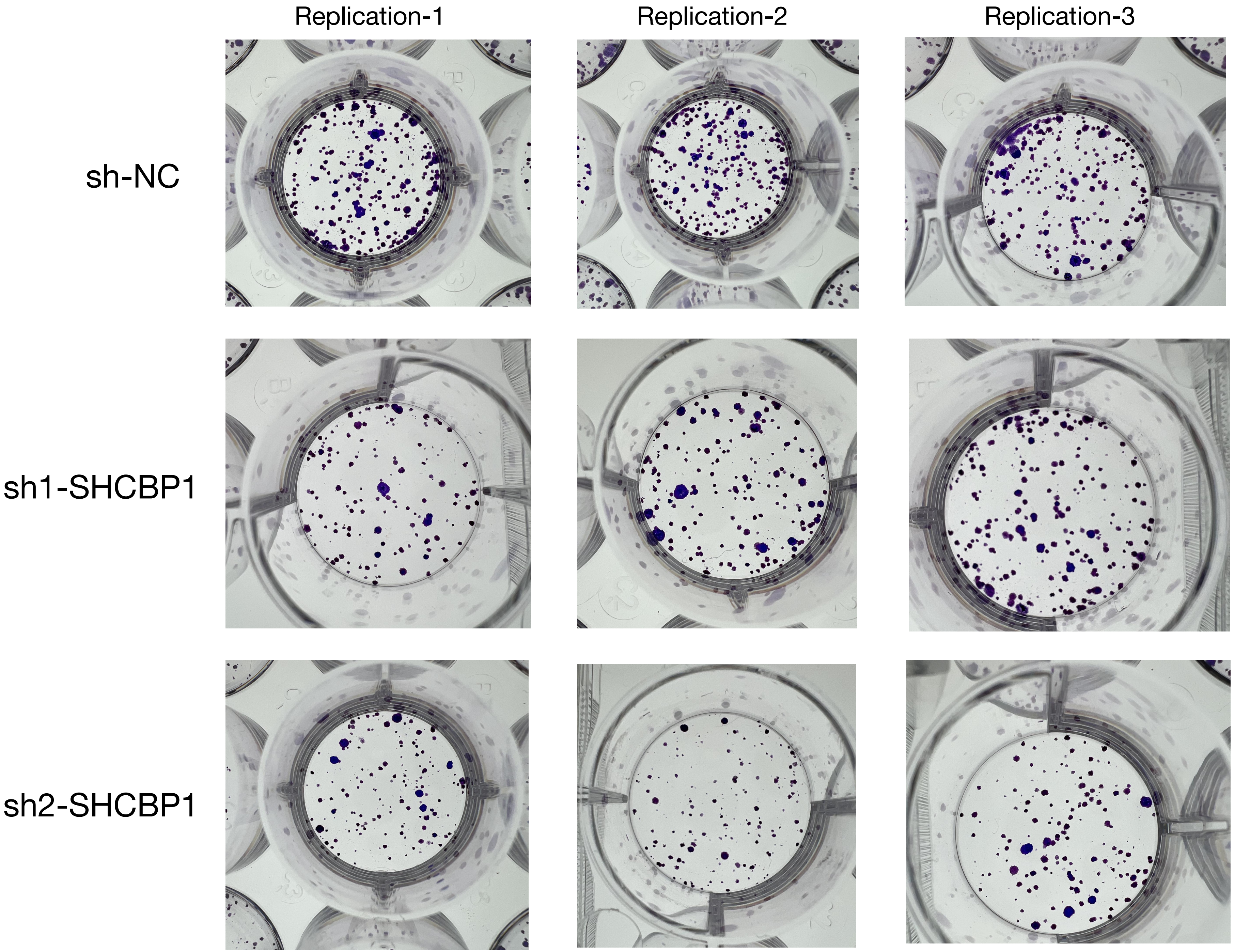
Replication-1
Replication-2
Replication-3
sh-NC
sh1-SHCBP1
sh2-SHCBP1
