# Supplementary material for: SHCBP1 Is Upregulated in Colon Adenocarcinoma and Promotes Tumor Cell Proliferation and Growth
Source: Curr Oncol. 2026 May 19;33(5):295. doi: 10.3390/curroncol33050295 (PMC13206487; doi:10.3390/curroncol33050295)
Supplement: Supplementary file 1 [file curroncol-33-00295-s001.zip › Figure legends S1-S2.pdf]

Figure S1. Quantitative analyses corresponding to Figure 1E–G.

(A) Densitometric quantification of SHCBP1 protein expression in COAD tissues and matched adjacent normal tissues corresponding to Figure 1E. Data are presented as mean ± SD.
(B) Quantitative analysis of SHCBP1 expression in the indicated COAD cell lines relative to NCM460 cells corresponding to Figure 1F. Data are presented as mean ± SD.
(C) Semi-quantitative analysis of SHCBP1 immunohistochemical staining scores in COAD tissues and matched adjacent normal tissues corresponding to Figure 1G. Data distribution is shown as a violin plot; median and interquartile range (IQR) are provided. Statistical comparison was performed using Wilcoxon matched-pairs signed-rank test.

Figure S2. Validation of SHCBP1 knockdown in HCT116 cells.

(A) Relative SHCBP1 mRNA expression levels measured by qPCR in HCT116 cells transfected with control shRNA (sh-NC) or SHCBP1-targeting shRNAs (sh1, sh2). Data are presented as mean ± SD from three independent experiments.
(B) Representative Western blot showing SHCBP1 protein expression after knockdown.
(C) Quantification of SHCBP1 protein levels from Western blot densitometry analysis. Data are presented as mean ± SD from three independent experiments.
(D) (D) Table showing raw Ct values and calculated relative expression for SHCBP1 knockdown experiments.
