# Supplementary material for: SHCBP1 Is Upregulated in Colon Adenocarcinoma and Promotes Tumor Cell Proliferation and Growth
Source: Curr Oncol. 2026 May 19;33(5):295. doi: 10.3390/curroncol33050295 (PMC13206487; doi:10.3390/curroncol33050295)
Supplement: Supplementary file 1 [file curroncol-33-00295-s001.zip › Figure S2.pdf]

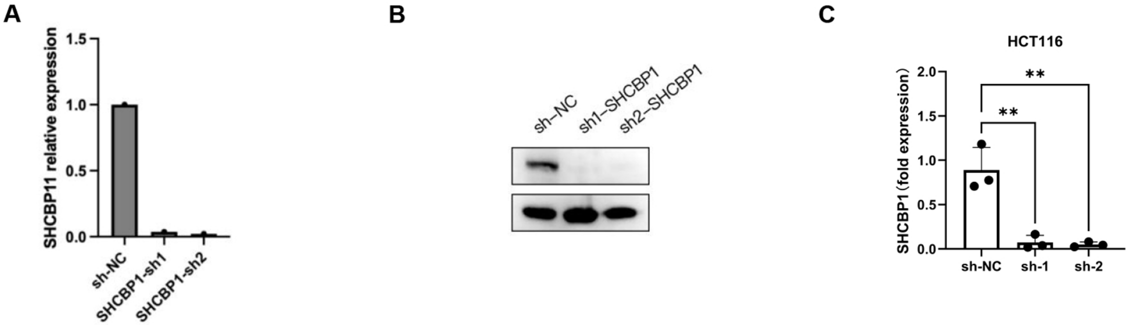

D

Group		Replicate 1	Replicate 2	Replicate 3	Mean	ΔCt (Target – HK)	ΔΔCt =ΔCt-ΔCt mean	2^-ΔΔCt
HCT116-sh-NC	Target Gene Ct	26.27	25.51	26.57	26.11666667	11.39	0	1
	House-keeping Gene Ct	14.61	14.55	15.02	14.72666667			
HCT16-sh1	Target Gene Ct	32.13	33.03	33.17	32.77666667	16.14333333	4.75333333	0.037076957
	House-keeping Gene Ct	17.42	16.03	16.45	16.63333333			
HCT116-sh2	Target Gene Ct	28.74	29.71	29.83	29.42666667	14.35	5.493333333	0.022199433
	House-keeping Gene Ct	14.97	15.06	15.2	15.07666667			
