# Supplementary material for: SHCBP1 Is Upregulated in Colon Adenocarcinoma and Promotes Tumor Cell Proliferation and Growth
Source: Curr Oncol. 2026 May 19;33(5):295. doi: 10.3390/curroncol33050295 (PMC13206487; doi:10.3390/curroncol33050295)
Supplement: Supplementary file 1 [file curroncol-33-00295-s001.zip › Table S1.pdf]

Table S1. Databases for Bioinformatics Analyses with Corresponding URLs

	Database	URL (accessed Jan-Mar 2023)
1	ONCOMINE	https://www.oncomine.org.
2	TIMER	https://cistrome.shinyapps.io/timer/
3	GEPIA2	http://gepia2.cancer-pku.cn
4	Human Protein Atlas	https://www.proteinatlas.org
5	UALCAN	http://ualcan.path.uab.edu
6	MethSurv	https://biit.cs.ut.ee/methsurv/
7	LinkedOmics	http://www.linkedomics.org
8	GeneMANIA	https://genemania.org
9	STRING	https://cn.string-db.org
