# Supplementary material for: SHCBP1 Is Upregulated in Colon Adenocarcinoma and Promotes Tumor Cell Proliferation and Growth
Source: Curr Oncol. 2026 May 19;33(5):295. doi: 10.3390/curroncol33050295 (PMC13206487; doi:10.3390/curroncol33050295)
Supplement: Supplementary file 1 [file curroncol-33-00295-s001.zip › Table S2.pdf]

Table S2. shRNA sequences and RT-qPCR primers

**Gene**	Sequence (5’-3’)
shRNA-1	GCTGTAGAGGGAATCTTAATT
shRNA-2	TAATGAAGAACTCGGATTTAT
SHCBP1-F	GCTACCGTGATAAACCAGGTTC
SHCBP1-R	AGGCTCTGAATCGCTCATAGA
GAPDH-F	GGAGCGAGATCCCTCCAAAAT
GAPDH-R	GGCTGTTGTCATACTTCTCATGG
