# Supplementary material for: SHCBP1 Is Upregulated in Colon Adenocarcinoma and Promotes Tumor Cell Proliferation and Growth
Source: Curr Oncol. 2026 May 19;33(5):295. doi: 10.3390/curroncol33050295 (PMC13206487; doi:10.3390/curroncol33050295)
Supplement: Supplementary file 1 [file curroncol-33-00295-s001.zip › Table S3.pdf]

**Table S3. The *p*-values for statistical tests in this manuscript.**

| Figure | Test Description | Group | *p*-value |
|---|---|---|---|
| Fig1B | SHCBP1 expression in TIMER database | Normal vs. Tumor | <0.001(The database does not provide specific *p*-values.) |
| Fig1C | SHCBP1 expression in GEPIA database | Normal vs. Tumor | <0.01(The database does not provide specific *p*-values.) |
| Fig1E | Relative expression of SHCBP1 | Normal vs. Tumor | 0.03 |
| Fig1H | K-M analysis based on HPA | Low vs. High expression | 0.15 |
| Fig1I | K-M analysis | Low vs. High expression | 0.0072 |
| Fig2A | Promoter methylation level of SHCBP1 in COAD | Normal vs. Primary | 3.45560247083654E-11（UALCAN database） |
| Fig2C | Survival analysis for CpG sites in COAD patients | cg20772904 | 0.08 |
| | | cg01377916 | 0.018 |
| | | cg02061047 | 0.17 |
| | | cg03693601 | 0.25 |
| | | cg04112058 | 0.58 |
| | | cg04540406 | 0.076 |
| | | cg05989100 | 0.57 |
| Fig5B | Number of colony formation | sh-NC vs. sh1-SHCBP1 | 0.0157 |
| | | sh-NC vs. sh2-SHCBP1 | 0.009 |
| Fig5D | CCK8 assays | 48h | <0.0001 |
| | | 72h | 0.00055 |
| Fig5F | Percentage of EDU positove cells | sh-NC vs. sh1-SHCBP1 | 0.0104 |
| | | sh-NC vs. sh2-SHCBP1 | 0.0099 |
| Fig5H | Tumor growth curves | 12day | 0.001 |
| | | 16day | <0.0001 |
