# Supplementary figures and images for: SHCBP1 Is Upregulated in Colon Adenocarcinoma and Promotes Tumor Cell Proliferation and Growth
Source: Curr Oncol. 2026 May 19;33(5):295. doi: 10.3390/curroncol33050295 (PMC13206487; doi:10.3390/curroncol33050295)

**A**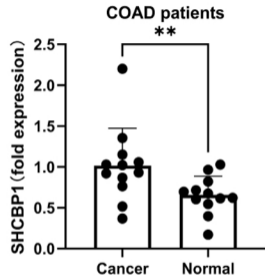**B**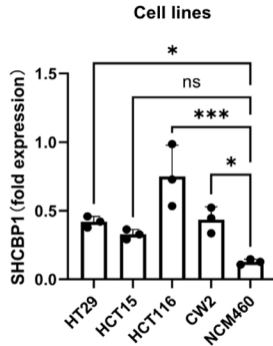**C**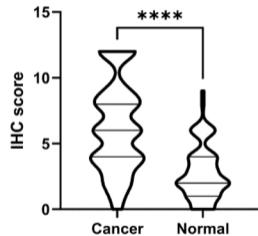

Supplement: Supplementary file 1 [file curroncol-33-00295-s001.zip › Figure S1.pdf]
